# Fecal Metabolome and Bacterial Composition in Severe Obesity: Impact of Diet and Bariatric Surgery

**DOI:** 10.1080/19490976.2022.2106102

**Published:** 2022-07-28

**Authors:** Nuria Salazar, Manuel Ponce-Alonso, María Garriga, Sergio Sánchez-Carrillo, Ana María Hernández-Barranco, Begoña Redruello, María Fernández, José Ignacio Botella-Carretero, Belén Vega-Piñero, Javier Galeano, Javier Zamora, Manuel Ferrer, Clara G de Los Reyes-Gavilán, Rosa Del Campo

**Affiliations:** a Department of Microbiology and Biochemistry of Dairy Products, Instituto de Productos Lácteos de Asturias (IPLA-CSIC), Villaviciosa & Diet, Microbiota and Health Group. Institute of Health Research of the Principality of Asturias (ISPA), Oviedo, Spain; b Department of Microbiology, Servicio de Microbiología. Hospital Universitario Ramón y Cajal, & Instituto Ramón y Cajal de Investigación Sanitaria (IRYCIS), & CIBERINFECT, Madrid, Spain; c Servicio de Endocrinología y Nutrición, Hospital Universitario Ramón y Cajal, & Instituto Ramón y Cajal de Investigación Sanitaria (IRyCIS), Madrid, Spain; d Instituto de Catálisis, Consejo Superior de Investigaciones Científicas, Madrid, Spain; e Servicios Científico-Técnicos, Instituto de Productos Lácteos de Asturias (IPLA-CSIC), Villaviciosa, Spain; f Department of Technology and Biotechnology of Dairy Products, Instituto de Productos Lácteos de Asturias (IPLA-CSIC), Villaviciosa & Molecular Microbiology Group, Institute of Health Research of the Principality of Asturias (ISPA), Oviedo, Spain; g Centro de Investigación Biomédica en Red de Fisiopatología de la Obesidad y Nutrición (CIBEROBN), Madrid, Spain; h Universidad de Alcalá, Madrid, Spain; i Grupo de Sistemas Complejos, Universidad Politécnica de Madrid, Spain; j Unidad de Bioestadística Clínica, Hospital Universitario Ramón y Cajal, & Instituto Ramón y Cajal de Investigación Sanitaria (IRYCIS), & CIBER Epidemiology and Public Health (CIBERESP), Madrid, Spain & Women’s Health Research Unit. Queen Mary University of London, London, UK; k Universidad Alfonso X El Sabio, Villanueva de la Cañada, Spain

**Keywords:** gut microbiota, bariatric surgery, metabolomic, SCFAs, machine learning for loss of weight excess prediction

## Abstract

The aim of this study was to monitor the impact of a preoperative low-calorie diet and bariatric surgery on the bacterial gut microbiota composition and functionality in severe obesity and to compare sleeve gastrectomy (SG) *versus* Roux-en-Y gastric bypass (RYGB). The study also aimed to incorporate big data analysis for the omics results and machine learning by a Lasso-based analysis to detect the potential markers for excess weight loss. Forty patients who underwent bariatric surgery were recruited (14 underwent SG, and 26 underwent RYGB). Each participant contributed 4 fecal samples (baseline, post-diet, 1 month after surgery and 3 months after surgery). The bacterial composition was determined by 16S rDNA massive sequencing using MiSeq (Illumina). Metabolic signatures associated to fecal concentrations of short-chain fatty acids, amino acids, biogenic amines, gamma-aminobutyric acid and ammonium were determined by gas and liquid chromatography. Orange 3 software was employed to correlate the variables, and a Lasso analysis was employed to predict the weight loss at the baseline samples. A correlation between Bacillota (formerly Firmicutes) abundance and excess weight was observed only for the highest body mass indexes. The low-calorie diet had little impact on composition and targeted metabolic activity. RYGB had a deeper impact on bacterial composition and putrefactive metabolism than SG, although the excess weight loss was comparable in the two groups. Significantly higher ammonium concentrations were detected in the feces of the RYGB group. We detected individual signatures of composition and functionality, rather than a gut microbiota characteristic of severe obesity, with opposing tendencies for almost all measured variables in the two surgical approaches. The gut microbiota of the baseline samples was not useful for predicting excess weight loss after the bariatric process.

## Background

Bariatric surgery is the most cost-effective treatment for reducing body mass index (BMI) in severe obesity (BMI >40 or >35 combined with comorbidities) and resolving endocrine-related dysfunctions such as type 2 diabetes mellitus (DM2) by both physiological and metabolic impact.^1,2^ The most common surgical approaches are Roux-en-Y gastric bypass (RYGB) and sleeve gastrectomy (SG), both by laparoscopy. Selecting the appropriate surgical approach requires a consensus between the patient and surgeon based on BMI, dietary habits, and comorbidities, and is not recommended in compulsive eating disorders.^3^ The physical reduction of the stomach forces drastic changes in the diet, and additional recommendations are made to maximize satiety also preserving the muscle mass, minimizing gastrointestinal symptoms, and enhancing weight loss. However, not all patients achieve complete loss of the baseline excess weight.

The gut microbiota’s contribution to obesity has been repeatedly discussed in recent years, but no solid conclusions have been reached, particularly for severe obesity. Bariatric surgery not only affects bacterial gut composition,^4,5^ with RYGB causing deeper changes, but also its overall functionality.^1,6^ Beyond the bacterial composition, scientific research is currently focused on microbial metabolism, particularly the mechanisms involved in DM2 remission,^7^ as well as on the microbiome functionality evolution,^8^ including short-chain fatty acids (SCFAs)^9^ and other microbial products related to the excess weight loss. The aim of the present study was to monitor the impact of a preoperative low-calorie diet and bariatric surgery on bacterial gut microbiota composition and functionality in severe obesity, to compare the RYGB and SG approaches, and to apply a Lasso predictive analysis based on multiple linear regressions to detect potential markers of excess weight loss.

## Patients and methods

### Study design and participants

A prospective study was conducted with 40 participants (22 women and 18 men, all older than 18 years) who were recruited during 2015–2017 at the Bariatric Surgery Unit of Ramón y Cajal University Hospital in Madrid, Spain. None of the participants reported infectious gastrointestinal disorders or antibiotic consumption in the last 3 months. Our center’s ethics committee approved the study (accession number 379/14), and all participants voluntarily signed an informed consent for participation in the study, which did not interfere with the bariatric surgical procedure. For RYGB, a small gastric bougie (15–30 ml) was created anastomosing to the jejunum by means of a Roux-en-Y assembly, with a biliopancreatic limb of 70–100 cm and an alimentary limb of 150 cm. For the highest BMIs, the limbs were lengthened up to 200 cm by decreasing the common channel. On SG, approximately 80% of the gastric volume was removed, leaving a bogie capacity of approximately 150–200 cc. The gastrectomy starts about 3–4 cm from the pylorus with linear mechanical staplers, and our clinical group usually reinforces the suture stapling line with a material from bovine pericardium (Peristrip-dry), obtaining very good results in terms of a low rate of leakage and bleeding from the stapling line.

Each participant provided 4 fecal samples at the following points: 1) baseline, the initial visit at the Bariatric Surgery Unit; 2) post-diet, after 1 month of the low-calorie diet; 3) 1 month after surgery; and 4) 3 months after surgery. Immediately after collection, the feces were aliquoted and frozen at −80°C until processing.

The baseline visit took place 1 month prior to the surgery to provide nutritional instructions with a high-protein low-calorie diet to reduce liver size in order to facilitate laparoscopic access (1400 calories for women and 1700 for men, distributed as 38% protein, 36% carbohydrates and 26% fat). Regardless of the surgical approach, the postoperative diet included only liquids for the first 2 days, followed by purées/soft food for the first month; thereafter, the patients received only general nutritional advice with no specific restrictions, with recommendations for a high protein intake (to prevent the loss of muscle mass) and multivitamin and mineral complexes. All participants were administered antibiotic prophylaxis during surgery with 2 g of intravenous amoxicillin/clavulanic acid.

### Fecal metabolome

The SCFA concentration was determined in cell-free fecal supernatants prepared as previously described^10^ using gas chromatography in a system composed of a 6890 N injection module (Agilent Technologies Inc., Palo Alto, CA, USA) connected to a flame injection detector and a mass spectrometry 5973 N detector (Agilent). Samples were analyzed in triplicate and the results were expressed as medians.

Quantification of amino acids, amines, ammonium and gamma-aminobutyric acid (GABA) was performed by ultra-high-performance liquid chromatography on cell-free fecal supernatants following diethyl ethoxymethylenemalonate (DEEMM, Sigma-Aldrich, USA) derivatization. Briefly, fecal supernatants were filtered through 3kDa centrifugal filters (Amicon Ultra-0.5, Merk KGaA, Germany), and DEEMM derivatization reactions were performed in 100 µl as previously described by Redruello et al.;^11^ however, this was the first time that the procedure was performed on feces. The accuracy of the chromatographic method employed to quantify all of the studied amino compounds was tested using a standard addition procedure.^12^ Thus, two known concentrations of analytes were independently added (in triplicate) to cell-free fecal supernatants. Non-spiked sample replicates (blanks) were used to determine the sample’s initial analyte content. The percentage recovery at each concentration was calculated as [(amount found in the spiked sample) – (amount found in the blank)/amount added] × 100. The recovery percentages for all analytes ranged from 80.1 to 104.4 when 0.2 mM was added (the accepted range for this concentration is 80–110) and from 90.1 to 104.3 when 1 mM was added (the accepted range is 90–107), reflecting the method’s good accuracy.^12^ Samples were also filtered through 0.22-µm-pore diameter polytetrafluoroethylene membranes (VWR International, USA) into conical vials (VWR) prior to injection into the ultra-high-performance liquid chromatography system.^11^

### Bacterial gut microbiota characterization

Samples were slowly thawed, first at −20°C for 24 h, followed by another 24 h at 4°C. After that, 0.5 g of the samples was solubilized in 5 ml of sterile water, centrifuged and extracted with the Speedtools tissue DNA extraction kit (Biotools, Madrid, Spain). The resulting total DNA was used for sequencing (2 × 300 bp) the V3 and V4 regions of the 16S rDNA gene on a MiSeq platform (Illumina, USA). To prepare the library, the 16S Metagenomic Sequencing Library Preparation protocol (Illumina; Cod. 15044223 Rev. A) was used with the following primers: Forward (5ʹTCGTCGGCAGCAGCGTCGTCAGATGTGTAAGACAGCCTACGGNGGCWGCAG), and Reverse (5ʹGTCTCGTGGCTCGAGGAGGTAAGAGAGACGACTACHVGGTATCTAATCC).

The quality of the raw sequences was measured according to the minimum length (250 bp), type of trimming quality measure (medium), quality value for trimming from the 3’ end (30) and trimming quality window (10 bp). Taxonomic affiliations were assigned using the SILVA 119 database and reads with a Ribosomal Database Project score <0.8 were assigned to the higher taxonomic rank, leaving the last rank as unidentified. The relative abundance and contingency tables of operational taxonomic units (OTUs) included taxa with very low representation. Sequences not assigned to any taxon or classified as non-bacterial were eliminated. Alpha and beta diversity studies were performed using the q2-diversity add-on of QIIME2,^13^ after normalizing the samples by rarefaction (subsampling without replacement). In addition, a linear effect size discriminant analysis (LEfSe)^14^ was performed to assess which taxa explained the differences between groups. Sequence data were deposited in Genbank (BioProject PRJNA639545).

### Statistical analysis

For the statistical analysis of the bacterial diversity results, we employed the q2-diversity plugin for QIIME2. In those groups/variables with statistically significant differences, we further explored which taxa explained these differences, using LEfSe, which employs the Kruskal–Wallis test and the pairwise Wilcoxon test. The alpha value for the Kruskal–Wallis factorial test was set at 0.05, and the linear discriminant analysis score threshold was set at 2.0.

The correlation between bacterial abundance and the various study metabolites was explored using a Big Data strategy, calculating the Spearman coefficient in Orange 3 software (https://orangedatamining.com) and the Wilcoxon test in GraphPad Prism 9 (GraphPad Software, USA, http://www.graphpad.com).

### Lasso regression model

To predict the excess weight loss at 3 months after surgery using only numerical variables from the baseline sample, we employed a Lasso regression model, a machine learning-based analysis with multiple linear regressions. The independent variable was the percentage of excess weight loss (%EWL), defined as the remaining excess weight 3 months after surgery divided by the excess weight in the baseline sample. The excess weight at time 2 (post-diet) and time 3 (1 month after surgery) were used as the dependent variables. Given that many dependent variables were analyzed and to avoid overfitting, the most important variables were delimited using the correlation between the main variable and the dataset of bacterial composition, SCFAs, amino acids and biogenic amines. The resulting dataset was divided into a test dataset (25%) and a training dataset (75%) to generate the Lasso model. The advantage of using this model is that it allows for a new selection of functions to obtain a better relationship between the test score and the training score. To measure the goodness of the method, the root mean square error was calculated. All calculations were performed using Python scripts developed by the authors (https://github.com/galeanojav/Severe_Obesity).

## Results

### %EWL

**Figure 1.**
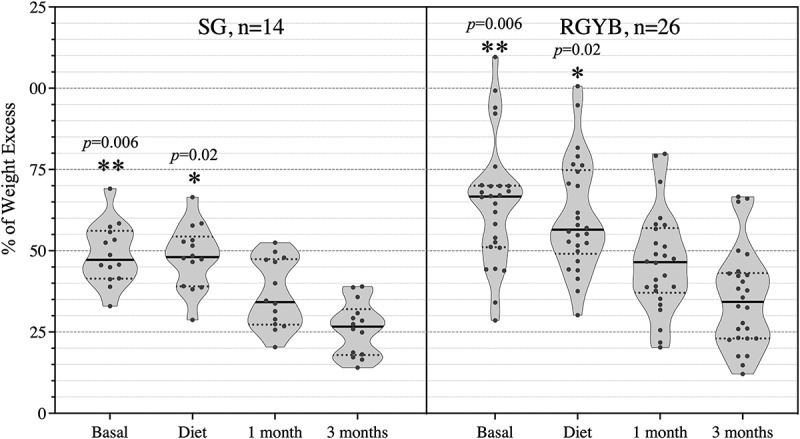
% Weight excess in the 40 patients along the 4 samples of each one. Statistically significant differences between SG and RYGB groups at basal and diet samples are marked as *p < .05, and **p > .01.

Table 1 summarizes the participants’ relevant clinical and demographic data, and Figure 1 provides an illustration of the %EWL progression. The RYGB group (n = 26) had significantly higher excess weight than the SG group (n = 14), both at baseline (57.2 vs. 47.3, *p* = .006) and after the diet (55.6 vs. 47.8, *p* = .02). In fact, the diet resulted in immediate weight loss only in the RYGB group; 3 months after surgery, however, the two groups had a similar %EWL (50.1% for RYGB and 49.0% for SG).

**Table 1.** Main characteristic of recruited severe obese (M = male, F = female).

Sex/Age (years)	T2DM/Resolution	Basal BMI	Weight Excess in Sample (kg) 1 2 3 4				Final % EWL
**SG (n = 14)**							
F/58		50.4	55.7	53.2	47.2	38.7	69.4
M/58		40.4	45.7	48.4	34.6	27.4	59.9
F/34		42.8	41.2	38.7	27.4	17.2	41.7
F/66		47.8	48.7	97.7	25.7	16.5	33.8
M/64	±	36.4	32.9	28.7	20.4	14.1	38.7
F/37		39.1	38.9	39.1	26.8	18.7	48.0
M/65	+/+	40.8	41.4	47.3	31.4	24.9	60.1
F/48		46.3	57.3	57.7	46.6	35.8	62.4
F/45		45.0	53.3	51.5	40.0	29.3	54.9
M/39		44.3	69.1	66.5	52.5	39.0	56.4
M/59		41.5	52.4	52.7	49.7	28.5	54.3
F/68		42.1	45.5	46.5	33.8	25.9	56.9
F/49		47.2	58.3	58.3	47.9	30.8	52.8
F/40		43.7	44.9	38.1	28.9	18.1	40.3
**Mean**		**43.1**	**47.3**	**47.8**	**33.8**	**23.5**	**50.1**
**RYGB (n = 26)**							
M/57		44.5	61.9	52.8	39.1	27.8	44.9
F/56	+/+	47.5	58.2	55.2	46.7	40.5	69.5
M/50		45.5	68.0	54.8	38.8	32.5	47.7
M/59	+/+	40.7	50.9	44.0	31.8	23.0	45.1
M/48		63.4	109.6	100.6	79.8	66.6	60.7
F/49		59.3	99.3	94.8	79.3	66.1	66.5
F/62		43.5	44.4	60.1	38.9	25.9	58.3
F/43	+/+	50.1	69.9	76.3	52.3	42.3	60.5
F/53		50.7	69.9	69.9	48.9	48.9	69.9
F/63	+/+	44.3	51.2	52.4	42.4	26.1	50.9
M/56	±	56.8	94.0	79.0	60.0	50.0	53.1
F/62	+/+	38.0	34.1	30.2	21.8	14.8	43.4
F/62	+/+	36.5	28.6	37.6	25.6	12.1	42.3
M/62		47.6	68.3	61.7	51.3	32.9	48.1
F/58		46.1	52.6	51.6	41.1	22.6	42.9
M/53		47.7	67.0	49.8	35.2	17.5	26.1
M/51		48.4	70.2	70.7	54.3	43.0	61.2
M/47		47.9	75.9	76.6	57.9	43.7	57.5
M/37		48.7	66.8	57.0	47.5	38.3	57.3
M/50	±	38.2	44.3	44.3	20.3	17.6	39.7
F/42		50.7	69.9	74.3	58.1	42.5	60.8
M/36	+/+	60.5	115.0	107.0	89.5	78.4	68.1
F/50		45.2	54.9	47.2	37.5	25.7	46.8
F/63		39.9	38.1	40.0	28.4	20.0	52.4
M/60	+/+	44.0	54.2	57.6	29.6	14.8	27.3
F/36		41.7	43.9	41.4	33.4	23.2	52.8
**Mean**		**46.3**	**57.2**	**55.6**	**39.9**	**27.3**	**49.0**

### Bacterial composition and distribution

The 160 fecal samples yielded a total of 11,889,557 reads corresponding to 250 OTUs. The data were normalized by establishing a sampling depth of 24,758 reads per sample.

**Figure 2.**
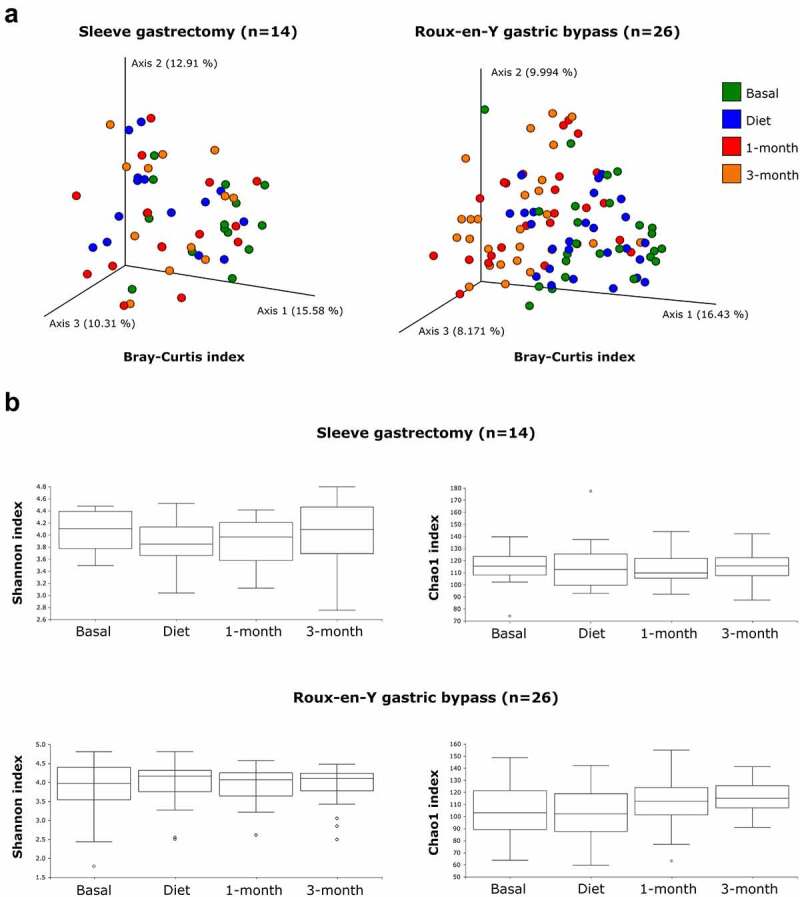
Bacterial diversity analysis in the two groups of patients throughout the 4 fecal samples. **A**: PCoA based on beta diversity values of all amplicon sequencing variants (Bray Curtis index), calculated from the bacterial profiles of stool samples. Each point represents a sample, and the color refers to sampling time. **B**: Alpha diversity values (Shannon and Chao1 indices).

Beta diversity based on the Bray-Curtis using all amplicon sequencing variants dissimilarity index was comparable for all patients at baseline and post-diet. From this point on, however, the progression of this index in the microbiota diverged for the two groups (Figure 2a), with significant changes in the SG group starting only after surgery (*p* = .03); these changes in SG patients were considerably less pronounced than for the RYGB group (*p* = .0015). No statistically significant differences in the alpha diversity values were detected by sampling point or type of surgery (Figure 2b). The Shannon index remained stable, but the Chao1 index experienced opposing trends for the SG (slight decrease) and RYGB (slight increase) groups, without reaching statistical significance in any case. There were no statistically significant correlations between alpha diversity and BMI (data not shown).

**Figure 3.**
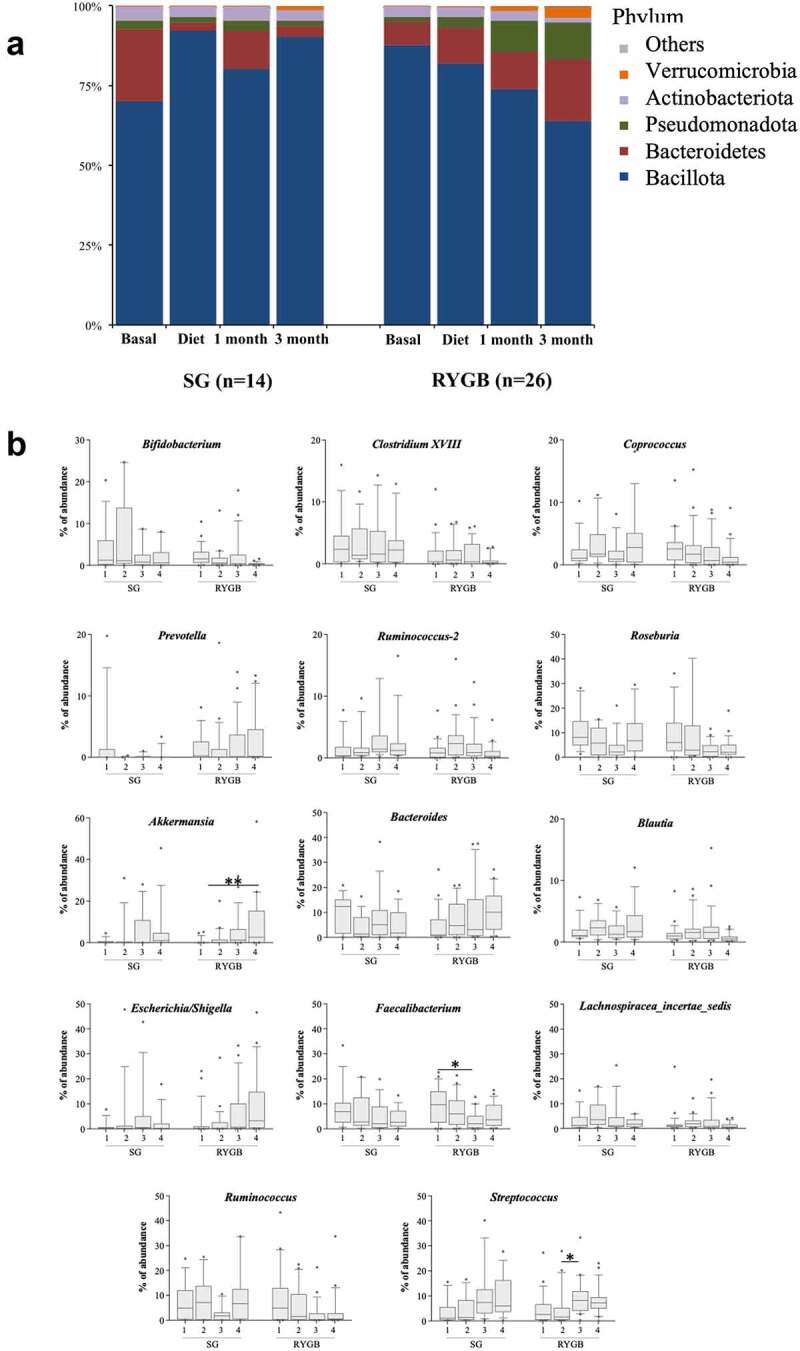
Bacterial distribution in feces. A: At phylum level. B: at genus level, showing only the most abundant (80% of the total abundance). Samples are represented as 1 = basal, 2 = diet, 3 = 1 month of surgery and 4 = 3 months of surgery. Statistically significant differences between samples are marked as *p < .05, and **p > .01.

The taxonomic analysis demonstrated the dominance of Bacillota (formerly Firmicutes) in the samples from both surgical groups (Figure 3a), with a total of 167 bacterial genera identified, 14 of which accounted for more than 80% of the overall abundance (Figure 3b). The differential traits in the microbiota of the SG group included the increase in Bacillota abundance across the bariatric process and the unique representation of certain minority genera such as *Butyricicoccus, Eggerthella, Gordonibacter* and *Intestinibacter* (data not shown).

In the SG group, there was a significant increase in certain genera of the phylum Bacillota (*Hungatella, Dorea* and *Eisenbergiella*) as the result of a decrease in the abundance of Bacteroidota after the diet. In contrast, the RYGB group showed a slight increase in the genera *Ruminococcus* and *Anaerococcus* and a decrease in the families *Lactobacillaceae* and *Bifidobacteriaceae*.

In terms of the surgical process, the intergroup differences were greater. Overall, bariatric surgery led to a decrease in the genera *Roseburia, Faecalibacterium, Ruminococcus* and *Bifidobacterium* and a significant increase in *Escherichia/Shigella* and *Akkermansia*.

**Figure 4.**
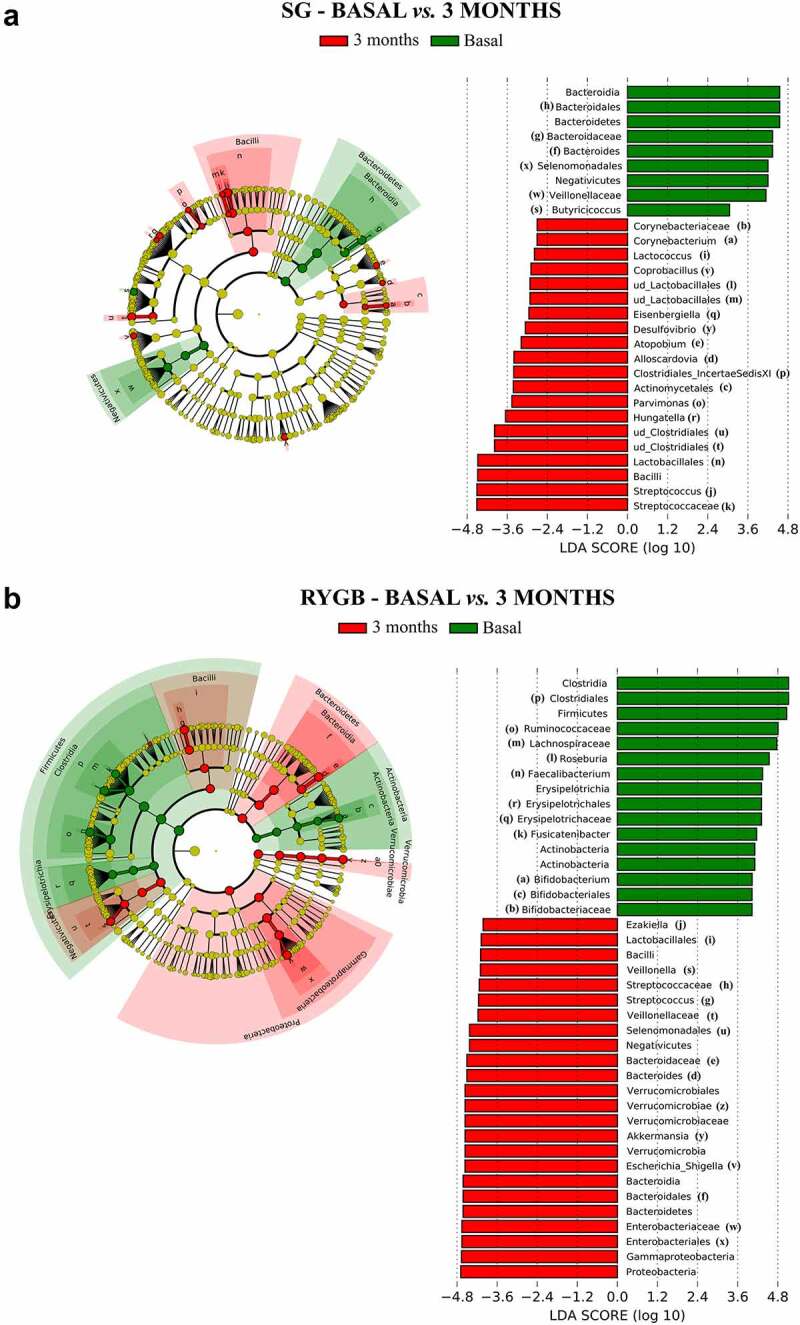
Bacterial taxa with differential abundance according to linear effect size discriminant analysis (LEfSe). The left side of each graph shows a cladogram, in which the yellow circles represent bacterial taxa that show no significant differences between the groups analyzed, while the green and red circles represent taxa whose abundance is significantly differential between the groups analyzed. The other side of each graph shows the significant taxa ordered according to the magnitude of the differences (LDA score). A: Comparison between baseline and 3 months after surgery in SG. B: Comparison between baseline and 3 months after surgery in RYGB (only taxa with LDA>4 are shown, to simplify the figure).

When stratifying according to type of surgery, the differences between the samples at baseline and at the end of the follow-up were much more profound in the RYGB group (Figure 4b), in accordance with the Bray-Curtis dissimilarities indexes. Thus, the Proteobacteria, Bacteroidota, Verrucomicrobiota and Fusobacteriota phyla experienced a significant increase in number at 3 months after RYGB surgery, inversely to Bacillota and Actinomycetota. However, the changes were considerably less marked in the SG group (Figure 4a), with a slight enrichment of certain Bacillota, such as *Streptococcus, Parvimonas, Hungatella, Lactobacillus* and *Desulfovibrio*, together with a decrease in Bacteroidota and Negativicutes.

**Figure 5.**
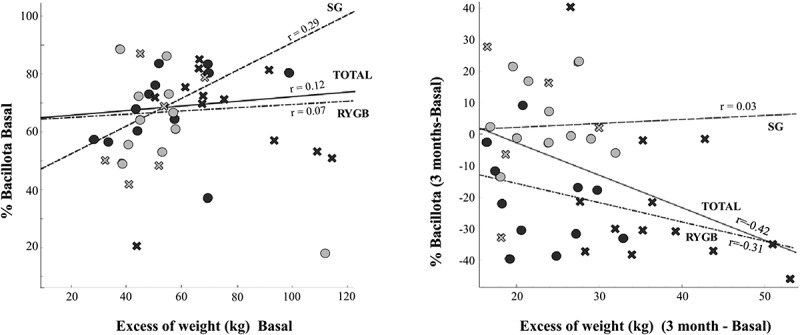
Correlation study between Firmicutes abundance and weight excess, distinguishing according to the type of surgery (SG: green; RYGB: red) and sex. Left: basal state, right: difference between 3 months after surgery and basal state.

The dynamics of the Bacillota phylum during the entire bariatric surgical process was particularly noteworthy for its opposing trends: the SG group maintained or even increased the high initial relative proportions, whereas the RYGB group experienced a marked decrease (Figure 3a). In the SG group, the sole correlation between weight and Bacillota abundance was the excess weight in the baseline sample (Figure 5, *r* = 0.29), whereas in the RYGB group, the %EWL was directly correlated to the decrease in Bacillota during the follow-up (*r* = −0.31).

### Short-chain and branched-chain fatty acids

**Figure 6.**
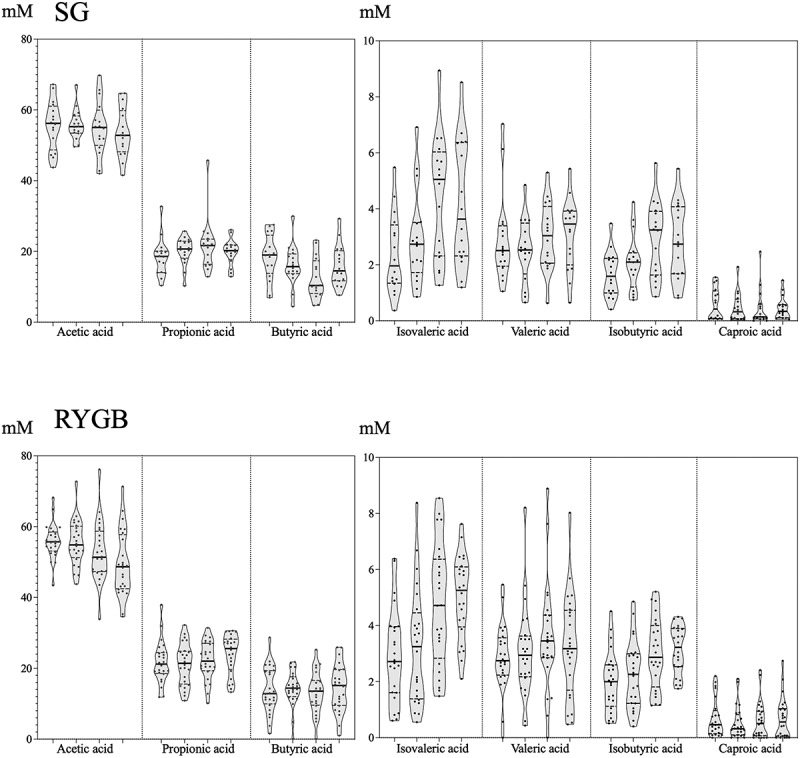
Evolution of the proportion (left) and absolute concentration (right, expressed in mM) of the majority (acetate, propionate and butyrate) and minority (caproate, isobutyrate, isovalerate and valerate) SCFA measured at the 4 times (from left to right: baseline, diet, 1 month after surgery, 3 months after surgery) for both groups. The horizontal line represents the mean of each distribution.

Table 2 and Figure 6 show the absolute concentrations and relative abundances of these microbial metabolites. The relative abundance of the main SCFAs (acetate >50%, propionate ~20% and butyrate 15%–20%) was comparable for the two groups. In general, there were higher concentrations of all measured SCFAs in the feces of the SG group, reaching statistical significance only for valeric acid (*p* = .04). There were no significant differences in SCFA and branched-chain fatty acid (BCFA) abundance according to sex, age or excess weight in the baseline sample (data not shown). The concentrations of each SCFA experienced moderate variations in the subsequent samples. The diet promoted a significant increase in the concentration of isobutyric acid, a branched SCFA, whereas no statistically significant changes were observed for the samples from the RYGB group. The postoperative fecal samples from the SG group had significantly decreased acetic and propionic acid concentrations compared with the concentrations after the diet. There was also a statistically significant decrease in acetic acid levels in the RYGB samples and a statistically significant increase in isobutyric acid levels. Statistically significant correlations for SCFA and BCFA and specific bacterial taxa were not detected.

**Table 2.** Median, standard deviation and range of SCFA values (mM) measured at the 4 times for both groups of patients.

Median ± SD Range (mM)	Acetate	Propionate	Butyrate	Isobutyrate	Valerate	Isovalerate	Caproate
SG*	**e**	**e**		**a**			**b**
Basal	83.1 ± 28.8 43.6–132.4	29.9 ± 10.5 6.9–44.2	29.9 ± 10.5. 6.9–44.2	2.3 ± 1.1 0.5–4.7	3.8 ± 2.3 0.7–10.3	2.8 ± 2.1 0.7–8.3	0.5 ± 0.9 0.08–3.4
Diet	92.1 ± 37.8 37.9–178.6	31.6 ± 17.9 8.3–82.8	31.6 ± 17.9 8.3–82.8	3.1 ± 1.0 0.7–4.5	4.0 ± 2.2 0.8–8.6	4.3 ± 1.5 1.4–6.2	0.5 ± 0.4 0.04–1.5
1 month	61.8 ± 42.2 10.4–168.5	25.0 ± 30.1 2.6–111.9	25.0 ± 30.1 2.6–111.9	2.5 ± 1.6 0.4–6.5	3.0 ± 2.6 0.4–10.4	3.9 ± 2.5 0.6–9.2	0.1 ± 0.3 0.0–0.8
3 months	62.6 ± 24.9 19.8–123.9	25.3 ± 9.6 3.9–40.7	25.3 ± 9.6 3.9–40.7	2.9 ± 1.3 1.0–5.7	3.6 ± 1.4 0.8–5.6	3.9 ± 2.3 1.5–8.6	0.3 ± 0.5 0.06–1.7
RYGB*	**b,c,e**			**c**		**c,e**	
Basal	69.1 ± 31.7 19.7–126.1	24.8 ± 12.3 9.7–52.3	17.2 ± 15.5 0.6–58.3	2.1 ± 1.0 0.2–4.3	3.2 ± 2.0 0.0–9.3	2.9 ± 1.5 0.9–6.8	0.7 ± 0.7 0.0–3.3
Diet	69.9 ± 33.0 22.8–158.2	23.5 ± 17.2 3.8–73.4	18.3 ± 14.8 0.0–66.7	2.1 ± 1.5 0.9–6.7	3.4 ± 2.8 0.4–14.2	2.8 ± 2.3 1.0–10.6	0.7 ± 0.6 0.0–2.4
1 month	53.6 ± 25.1 14.2–114.9	21.7 ± 13.1 3.1–51.2	10.5 ± 10.2 0.0–35.2	2.4 ± 1.1 0.5–5.7	3.3 ± 2.4 0.0–11.7	3.6 ± 1.7 1.6–9.0	0.5 ± 0.4 0.0–1.6
3 months	52.9 ± 22.9 16.0–91.1	26.5 ± 15.8 3.6–55.7	16.1 ± 14.9 0.9–56.4	3.0 ± 1.5 0.9–8.7	2.9 ± 2.1 0.6–7.8	4.7 ± 2.8 1.4–15.7	0.4 ± 0.6 0.0–3.1

Notes: *a statistically significant difference determined by Wilcoxon test between baseline and diet; b: baseline and 1 month; c: baseline and 3 months; d: diet and 1 month; and e: diet and 3 months. Only values of p ≤ 0.05 were considered.

### Amino acids, biogenic amines and GABA

Fecal concentrations of amino acids and biogenic amines are shown in Tables 3 and 4, respectively. A comprehensive analysis was performed to detect correlations between each bacterial taxa abundance and the proportion of amino acids, biogenic amines and GABA, without success. Significant differences were observed between bariatric surgery groups, as well as different trends throughout the study period within each group. Globally, higher concentrations of amino acids were found in the SG samples both after the diet and after surgery, while the biogenic amines cadaverine, histamine and tyramine had significantly higher concentrations in fecal samples of the group of RYGB patients.

**Table 3.** Median, standard deviation and range of amino acid values (mM) measured at the 4 times for both groups of patients.

Median ± SD Range (mM)	Ala	Arg	Asn	Asp	Gln	Glu	Gly	His	Ile	Leu	Lys
SG*	**a,c**	**a**			**a,c**	**a**			**c**	**c**	
Basal	4.2 ± 3.8 0.8–15.5	0.3 ± 0.3 0.06–1.3	0.03 ± 0.2 nd–1.1	1.3 ± 0.9 0.4–3.7	0.1 ± 0.2 nd–0.9	3.5 ± 3.6 1.0–14.9	1.2 ± 1.5 0.2–6.5	0.1 ± 0.1 0.04–0.5	0.9 ± 1.0 0.05–3.6	1.5 ± 1.5 0.05–5.3	2.0 ± 1.3 0.05–5.0
Diet	8.2 ± 5.2 1.6–20.5	0.4 ± 0.3 0.08–1.2	0.02 ± 0.1 nd–0.7	1.0 ± 0.6 0.6–2.9	0.2 ± 0.1 0.01–0.4	7.3 ± 3.0 0.2–12.1	1.8 ± 1.9 0.4–7.1	0.2 ± 0.2 0.03–0.8	1.4 ± 1.3 0.2–5.1	3.2 ± 1.7 0.5–6.7	2.6 ± 1.5 0.8–5.8
1 month	6.5 ± 5.2 1.6–24.4	0.2 ± 0.2 0.02–0.7	0.02 ± 0.04 nd–0.1	1.1 ± 1.0 0.2–3.8	0.1 ± 0.2 0.08–1.1	4.9 ± 3.6 1.3–16.7	1.9 ± 2.3 0.4–10.5	0.1 ± 0.1 0.03–0.6	1.8 ± 1.1 0.3–4.6	3.2 ± 1.9 0.4–8.8	2.2 ± 1.3 0.1–5.5
3 months	8.3 ± 5.3 2.7–19.8	0.2 ± 0.2 0.03–0.8	0.03 ± 0.04 nd–0.1	1.1 ± 0.6 0.4–3.1	0.1 ± 0.1 nd–0.5	4.7 ± 2.4 2.2–10.2	2.4 ± 1.9 0.6–6.8	0.2 ± 0.1 0.01–0.6	2.2 ± 1.2 0.5–4.6	3.1 ± 1.8 1.1–7.3	2.6 ± 1.2 0.7–4.7
RYGB*		**a,f**			**a,f**	**a,e,f**					
Basal	4.3 ± 2.9 1.5–14.0	0.5 ± 0.2 0.09–0.9	0.02 ± 0.04 nd–0.1	1.1 ± 0.6 0.3–2.6	0.1 ± 0.1 0.03–0.5	5.2 ± 2.5 1.3–11.9	1.2 ± 2.3 0.2–10.9	0.1 ± 0.4 0.03–1.9	0.9 ± 0.7 0.3–3.5	1.5 ± 1.2 0.8–6.7	1.8 ± 0.7 0.4–3.6
Diet	5.2 ± 4.1 0.8–17.9	0.3 ± 0.2 0.02–0.9	nd±0.03 nd–0.1	1.3 ± 0.7 0.2–2.9	0.1 ± 0.1 nd–0.7	4.8 ± 3.0 0.6–12.2	1.7 ± 1.3 0.1–5.7	0.1 ± 0.1 0.03–0.4	1.3 ± 0.9 0.1–4.0	2.3 ± 1.4 0.2–6.1	1.8 ± 1.0 0.4–4.8
1 month	4.1 ± 3.8 0.9–15.6	0.2 ± 0.1 0.02–0.6	0.01 ± 0.2 nd–1.2	0.8 ± 0.6 0.2–3.1	0.1 ± 0.2 0.01–1.1	3.7 ± 2.5 nd–9.2	1.2 ± 0.9 0.2–3.2	0.1 ± 0.1 0.03–0.8	0.9 ± 1.4 0.1–7.4	1.8 ± 2.3 0.3–12.2	1.4 ± 1.0 0.5–4.1
3 months	3.0 ± 4.1 1.0–18.6	0.5 ± 0.4 0.04–2.1	nd±0.02 nd–0.07	0.9 ± 0.7 0.1–3.5	0.1 ± 0.08 0.01–0.2	2.2 ± 2.1 0.5–8.6	0.7 ± 1.1 0.2–5.5	0.1 ± 0.09 0.01–0.4	0.6 ± 1.0 0.1–3.9	1.3 ± 1.7 0.4–7.3	1.2 ± 1.0 0.5–4.6
Median ± SD Range (mM)	**Met**	**Phe**	**Pro**	**Ser**	**Thr**	**Trp**	**Tyr**	**Val**	**Orn**	**BCAAs**	**TOTAL amino acids**
SG*		**c**	**c,e**					**c**		**c**	
Basal	0.2 ± 0.3 0.02–1.0	0.7 ± 0.5 0.05–2.0	0.09 ± 0.5 0–1.9	1.0 ± 1.0 nd–3.7	1.4 ± 1.9 0.1–8.5	0.1 ± 0.1 0.05–0.6	0.9 ± 0.7 0.1–2.7	1.2 ± 1.0 0.1–4.0	0.2 ± 0.3 0.05–1.5	3.5 ± 3.4 0.4–13.1	
Diet	0.6 ± 0.4 0.01–1.5	1.4 ± 0.6 0.3–2.4	0.1 ± 0.2 0–0.6	1.5 ± 1.1 nd–3.9	1.6 ± 2.8 0.3–11.4	0.1 ± 0.1 0.05–0.4	1.8 ± 0.8 0.4–3.5	1.9 ± 1.2 0.3–4.8	0.3 ± 0.3 0.1–1.2	6.5 ± 4.3 1.2–16.7	
1 month	0.3 ± 0.4 0.03–1.8	1.1 ± 0.6 0.1–2.9	0.03 ± 0.2 0.002–0.9	1.8 ± 0.8 0.3–3.5	1.5 ± 3.0 0.3–10.5	0.2 ± 0.1 0.03–0.7	1.4 ± 0.8 0.2–3.6	2.0 ± 1.4 0.3–6.3	0.3 ± 0.2 0.08–0.9	6.7 ± 4.5 1.1–19.7	
3 months	0.4 ± 0.3 0.02–1.3	1.3 ± 0.5 0.5–2.6	0.04 ± 0.1 0–0.5	1.6 ± 0.8 0.7–3.5	1.7 ± 0.9 0.5–3.7	0.2 ± 0.08 0.08–0.4	1.2 ± 0.6 0.6–3.0	2.2 ± 1.3 0.6–4.7	0.4 ± 0.2 0.2–1.0	7.7 ± 4.3 2.5–15.9	
RYGB*				**a**			**a**				
Basal	0.2 ± 0.2 nd–1.2	0.6 ± 0.5 0.3–3.1	0.05 ± 0.1 nd–0.8	0.9 ± 0.4 0.3–2.6	1.1 ± 0.6 0.4–3.0	0.1 ± 0.2 0.05–1.0	0.7 ± 0.2 0.4–1.7	0.9 ± 0.9 0.4–4.5	0.2 ± 0.6 0.08–3.3	3.5 ± 2.9 1.6–14.7	
Diet	0.3 ± 0.2 0.03–1.0	0.9 ± 0.4 0.1–1.9	0.04–0.1 nd–0.6	1.3 ± 0.9 0.1–3.1	1.2 ± 0.8 0.1–2.6	0.1 ± 0.1 0.01–0.5	0.8 ± 0.5 0.1–1.9	1.5 ± 1.3 0.1–6.0	02 ± 0.2 0.04–0.7	5.5 ± 3.8 0.4–14.7	
1 month	0.2 ± 0.7 0.02–3.7	0.6 ± 0.8 0.1–4.3	0.1 ± 0.3 nd–1.1	1.0 ± 0.7 nd–3.0	0.8 ± 2.0 0.2–10.5	0.1 ± 0.3 0.02–1.5	0.6 ± 0.4 0.2–2.0	1.0 ± 2.1 0.2–10.8	0.3 ± 1.5 0.09–7.6	3.8 ± 5.9 0.7–30.5	
3 months	0.2 ± 0.3 1.0–18.6	0.7 ± 1.4 0.2–6.0	0.07 ± 0.4 nd–1.8	0.8 ± 0.7 0.2–3.0	0.7 ± 0.7 0.2–2.9	0.1 ± 0.1 0.02–0.5	0.6 ± 0.5 0.2–2.5	0.7 ± 1.3 0.2–5.5	0.2 ± 0.3 0.08–1.3	2.6 ± 4.0 0.8–16.5	

Abbreviations: Ala: alanine, Arg: arginine, Asn: asparagine, Asp: aspartic acid, Gln: glutamine, Glu: glutamate, Gly: glycine, His: histidine, Ile: isoleucine, Leu: leucine. *a: statistically significant difference determined by Wilcoxon test between baseline and diet; b: baseline and 1 month; c: baseline and 3 months; d: diet and 1 month; e: diet and 3 months; and f: 1 month and 3 months. nd: not detected. Only values of p ≤ 0.05 were considered. Lys: lysine, Met: methionine, Phe: phenylalanine, Pro: proline, Ser: serine, Thr: threonine, Trp: Tryptophan, Tyr: tyrosine, Val: valine, Orn: ornithine, BCCAs: Branched-Chain Amino Acids (leucine, isoleucine and valine). *a: statistically significant difference determined by Wilcoxon test between baseline and diet; b: baseline and 1 month; c: baseline and 3 months; d: diet and 1 month; e: diet and 3 months; and f: 1 month and 3 months. nd: not detected. Only values of p ≤ 0.05 were considered.

**Table 4.** Median, standard deviation and range of the values of biogenic amines, phenylethylamine (PEA) and γ–aminobutyric acid (GABA), and ammonium (mM).

Median ± SDRange (mM)	Agmantine	Histamine	Tiramine	Putrescine	Tryptamine	Cadaverin	PEA	GABA	Ammonium
SG*			**e**		**f**			**e,f**	**e,f**
Basal	0.03 ± 0.09 nd–0.4	0.07 ± 0.09 nd–0.3	0.01 ± 0.02 nd–0.1	0.3 ± 0.5 0.03–1.7	nd±0.03 nd–0.1	0.5 ± 1.6 0.01–6.2	nd±0.01 nd–0.05	0.2 ± 1.9 0.02–6.6	32.7 ± 11.3 7.5–40.3
Diet	0.02 ± 0.1 nd–0.6	0.05 ± 0.1 nd–0.5	0.01 ± 0.04 nd–0.1	0.1 ± 0.3 0.02–1.3	nd±0.01 nd–0.04	0.6 ± 1.4 nd–4.0	nd ± nd nd–0.002	0.3 ± 1.2 0.03–3.5	34.0 ± 16.5 10.1–63.5
1 month	0.01 ± 0.03 nd–0.1	0.02 ± 0.3 nd–1.3	nd±0.1 nd–0.5	0.06 ± 0.9 nd–3.9	nd ± nd nd–0.03	0.5 ± 2.0 nd–7.1	nd ± nd nd–0.004	0.5 ± 0.7 0.03–2.3	20.7 ± 15.4 5.9–71.1
3 months	0.02 ± 0.04 nd–0.1	0.02 ± 0.1 nd–0.5	0.01 ± 0.02 nd–0.1	0.01 ± 0.4 0.01–1.6	nd±0.02 nd–0.1	0.4 ± 1.8 nd–6.3	0 ± 0 0–0.01	0.4 ± 1.4 0.04–5.0	24.6 ± 13.9 6.6–53.3
RYGB*					**a**			**a**	
Basal	0.02 ± 0.07 nd–0.3	0.07 ± 0.2 nd–1.1	0.03 ± 0.3 nd–1.5	0.2 ± 0.7 0.02–2.0	nd ± nd nd–0.004	3.0 ± 2.3 nd–7.7	nd ± nd nd–0.02	0.2 ± 1.5 0.02–7.2	27.9 ± 13.6 6.9–60.4
Diet	0.02 ± 0.02 nd–0.07	0.08 ± 0.1 nd–0.5	0.02 ± 0.1 nd–0.5	0.1 ± 0.9 nd–4.7	nd ± nd nd–0.01	1.4 ± 2.0 0.1–6.7	nd ± nd nd–0.01	0.1 ± 1.7 nd–6.8	28.6 ± 13.0 6.5–47.9
1 month	0.02 ± 0.04 nd–0.1	0.04 ± 0.1 nd–0.5	0.05 ± 0.4 nd–2.1	0.4 ± 0.8 nd–3.1	nd ± nd nd–0.03	2.9 ± 3.7 0.01–12.8	nd±0.04 nd–0.2	0.2 ± 0.2 0.03–0.7	30.7 ± 12.5 9.6–57.1
3 months	0.01 ± 0.05 nd–0.2	0.1 ± 0.1 nd–0.8	0.1 ± 0.5 nd–0.2	0.6 ± 0.7 nd–2.3	nd ± nd nd–0.01	3.1 ± 3.0 nd–11.4	nd ± nd nd–0.02	0.7 ± 1.8 0.0–8.0	40.5 ± 12.5 11.5–58.7

*Notes: a: statistically significant difference determined by Wilcoxon test between baseline and diet; b: baseline and 1 month; c: baseline and 3 months; d: diet and 1 month; e: diet and 3 months; and f: 1 month and 3 months. Only values of p ≤ 0.05 were considered

There was a statistically significant increase in alanine, arginine, glutamine and glutamic acid levels after the low-calorie diet in the SG group, as well in the levels of the biogenic amine tryptamine and the neurotransmitter GABA. Regarding the impact of the surgical process, there was a statistically significant increase in alanine and branched amino acid levels and a decrease in phenylalanine, proline and glutamine levels in the SG group when comparing the baseline samples and the samples taken 3 months after surgery.

By contrast, the diet produced an increase in the RYGB group in the concentrations of tyrosine and serine and a decrease in arginine, glutamine and glutamic acid levels. In addition, surgery was associated with a statistically significant decrease in glutamic acid levels and a statistically significant increase in levels of the biogenic amine tryptamine and GABA.

### Ammonium

**Figure 7.**
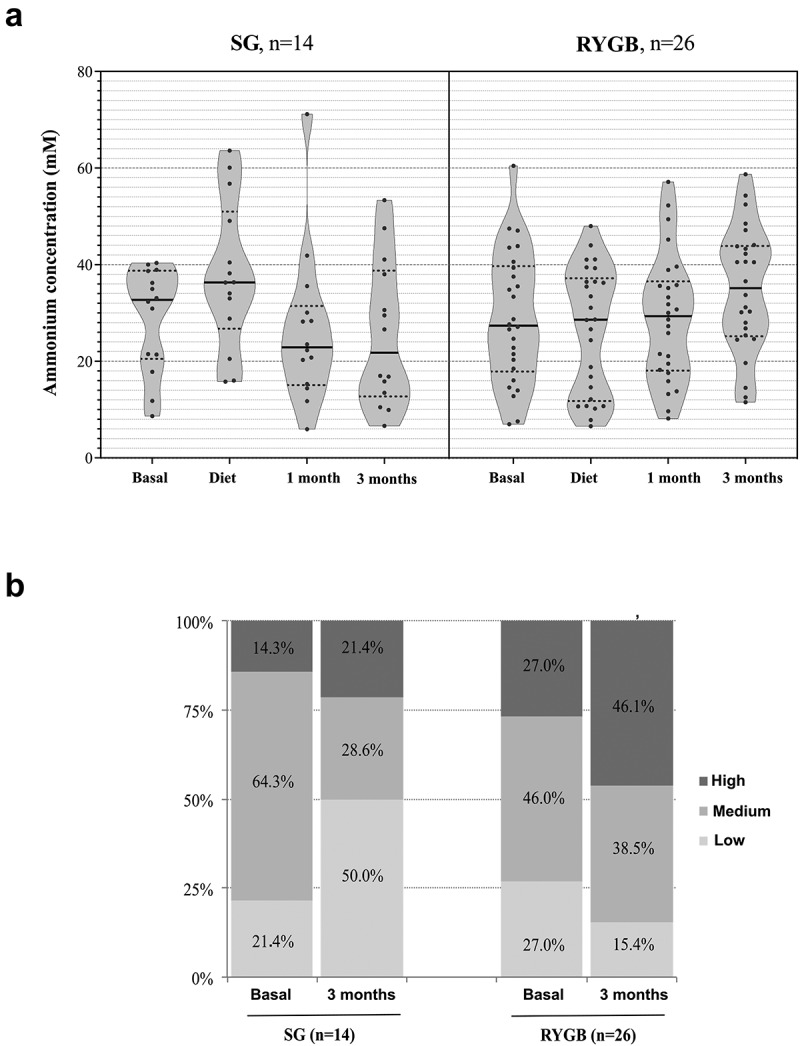
Results of fecal ammonium determination. A: ammonium concentration (mM) at the 4 fecal samples, for each group of patients. B: Percentage distribution of patients according to their fecal ammonium levels at the basal sample and at the end (3 months) of follow–up, for each type of surgery.

The progression of the fecal ammonium concentrations showed opposing trends over the sampling time for the two surgical groups, with a significant decrease for the SG group and a significant increase for the RYGB group (Figure 7a). Given that the normal range for fecal ammonium has not yet been defined, we arbitrarily established three categories based on the following tertiles: low (0–19 mM), medium (20–39 mM) and high concentration (>40 mM) (Figure 7b). We compared the bacterial abundance in the baseline samples to the fecal ammonium profile, demonstrating the predominance of *Paraprevotella* to the detriment of other members of the Bacteroidales order (*Parabacteroides, Barnesiella* and *Odoribacter*) and *Lentisphaerae* in those patients with higher ammonium levels (Supplementary Figure 2).

### DM2 resolution after bariatric surgery

During enrollment, 12 participants (7 men; 10 of them undergoing RYGB) had insulin-dependent DM2. After the surgery, 9 (75%, 8 of them undergoing RYGB) of the 12 experienced complete remission without drug treatment, while the remaining 3 patients continued to require oral metformin and insulin.

The differential bacterial abundance was analyzed in the 8 patients who underwent RYGB with complete resolution of the diabetes (baseline vs. 3-month samples), showing that the phyla Verrucomicrobiota (*Akkermansia*) and Fusobacteriota (*Fusobacterium*) increased significantly after surgery, while the relative abundance of the phyla Bacillota (*Faecalibacterium, Erysipelotrichia, Gemmiger* and *Lactobacillus*) and Actinobacteriota (*Bifidobacterium*) decreased. When comparing these 8 patients with the remaining 18 RYGB patients at baseline, a significantly lower abundance of the Clostridia class was associated with DM2, although surgery resulted in a clear decrease of this taxon in both groups (Supplementary Figure 3).

### Lasso model

Analyses with the Lasso model failed to identify any metabolite able to predict %EWL after 100 repetitions; however, they were able to identify 8 minority bacterial genera (Table 5). The root mean square error values were always low (approximately 0.05). The model pointed to *Anaerofustis* and *Acetanaerobacterium* as the most discriminant genera; however, both are underrepresented in the global gut microbiota. The most promising finding was related to *Bifidobacterium*, a genus considerably more abundant in the microbiota than the others, despite its lower prediction capability. This genus decreased in both groups of surgery as directly correlated with weight loss.

**Table 5.** Median values of the 100 Lasso regression values obtained for the significant bacterial genera and their abundance at baseline in each group of patients.

Genera	Lasso regression	% Basal abundance SG RYGB	
*Anaerofustis*	**4.910**	0.001	0.001
*Acetanaerobacterium*	**3.234**	0.002	0.01
*Coprobacter*	0.182	0.02	0.03
*Anaerophylum*	0.088	0.002	0.002
*Slackia*	0.024	0.07	0.05
*Gemmiger*	0.003	1.7	2.5
*Romboutsia*	0.002	0.3	0.1
*Bifidobacterium*	0.001	3.3	2.4

## Discussion (BRAY Curtis!)

Obesity is a complex and multifactorial disease that goes far beyond simple excess weight, requiring an individualized study to understand its nature, causes, and treatment, particularly in severe obesity. The gut microbiota’s contribution to obesity is gradually being recognized, although the lack of established normal ranges for the microbiota’s composition and functionality precludes reaching solid conclusions in certain cases. Most of the available studies have been designed to identify significant differences in bacterial gut microbiota composition between lean individuals and those with obesity or to decipher the impact of each surgical approach on bacterial composition, employing a wide range of methodologies and analytical strategies.

The present study aimed to monitor fecal microbiota composition and functionality fluctuations during the bariatric process in severe obesity and included robust statistical analysis with machine learning approaches for prediction. Our main findings are the description of fecal profiles of SCFAs, amino acids and biogenic amines in severe obesity during the bariatric process, highlighting the comparatively high levels of ammonia detected in the patients who underwent RYGB compared with those who underwent SG and, relevantly, the suggestion that a particular baseline gut microbiota might be necessary to reach the highest BMI values, as the dynamics of Bacillota suggests.

Previous studies have emphasized the low microbial gene richness, low alpha diversity values and higher abundance of Bacillota in severe obesity.^6,15–18^ The utility of the Bacillota/Bacteroidota ratio (formerly the Firmicutes/Bacteroidetes ratio) in obesity is controversial, and recent data point to its lack of validity as a microbiota marker, given that an expansion of Bacillota leads to the proportional reduction of the remaining phyla.^19,20^ The most noticeable feature we found was that, although the Bray-Curtis analysis found no significant difference in phyla abundance in the baseline samples, there was a direct correlation between Bacillota abundance and baseline BMI occurring in SG, whereas in RYGB, the extremely high proportions of Bacillota in the baseline samples experienced a proportional decrease in accordance with the %EWL. Surprisingly, the abundance of Bacillota increased in the postoperative SG samples with no correlation to weight loss.

The dissimilar impact of the two bariatric surgical approaches on gut microbial composition has been exhaustively reviewed, with consistent results.^9,21–27^ Alpha diversity dynamics appear not to be reproducible across studies; however, beta diversity analyses consistently find deeper changes for RYGB, including an expansion of the Pseudomonadota (formerly Proteobacteria) phyla.^28–33^ Biliary acid circuit redistribution, whose antibacterial activity limits the expansion of gamma-Proteobacteria in the small intestine, is likely the major cause of the increase in members from this phylum in samples from patients who undergo RYGB. Despite the differential trends in bacterial composition, weight loss was uniformly reached in both groups.

Acetic, propionic and butyric acids were the most abundant SCFAs in our fecal samples, as other authors have reported.^34^ SCFAs are derived mainly from carbohydrate bacterial fermentation and a small amount is also produced by the colonic fermentation of the branched-chain amino acids valine, leucine, and isoleucine, leading to the production of the BCFAs isobutyric, isovaleric and 2-methyl butyric acid.^9,35^ A number of authors have recently reported an increase in fecal BCFAs after bariatric surgery.^33,36^ In our study, only the increase in isobutyric acid levels was statistically significant in the RYGB group after surgery with the concomitant decrease of leucine and valine levels. In general, we observed modest variations in SCFAs concentrations, highlighting the decrease in acetic acid levels in both groups; however, the bariatric process implies drastic changes in food intake, particularly after surgery. SCFAs can represent up to 10% of the total energy intake, and it has been postulated that there is a significant overproduction of these microbial metabolites in obesity,^18,37^ particularly propionic acid.^38^ In general, the major limitation to reaching solid conclusions is the lack of defined cutoffs for SCFAs, as well as measurement standardization for food intake and the subsequent time required for SCFAs production.

We performed an exhaustive analysis to decipher the relationship between bacterial taxonomic levels and SCFA concentrations but did not obtain relevant results. In that sense, certain metabolic routes of SCFA production could be shared by unrelated bacterial taxa, which, together with the possible absorption by the intestinal epithelium of formed compounds, makes it difficult to establish clear correlations between fecal SCFA levels and bacterial abundance.^34,35,39^

Most dietary proteins are absorbed during digestion, and only a small proportion of undigested proteins (approximately 5%), together with the rest of the luminal apoptotic host cells or cells from dead microorganisms, reach the colon. There is currently a lack of data on the normal concentrations of amino acids in feces, even more so in the case of bariatric surgery, which again limits to extract sound conclusions from our analysis; significant increases in glutamate levels have been reported only in rats subjected to RYGB.^40^ We found noticeable variations in the levels of certain amino acids associated with the preoperative low calorie diet and the type of bariatric surgery. Notably, levels of total amino acids were higher in the samples from the SG group than from the RYGB group after the low calorie diet and surgery, whereas conversely, certain biogenic amines had higher levels in the RYGB samples. Bariatric surgery, particularly RYGB by malabsorption, could lead to increased protein concentrations in the lumen of the colon. Amino acids from these proteins could be metabolized by anaerobic bacteria, increasing the potential production of toxic metabolites. The higher levels of biogenic amines found in RYGB support this conclusion, and although the low fecal concentrations of these compounds are not suggestive a priori of endogenous intoxication, higher microbial production, particularly in diseases involving intestinal dysmotility, cannot be ruled out.^41^

One of the most striking results of our study was the opposing tendency in fecal ammonium concentrations of the two approaches. RYGB provoked an abrupt increase (almost doubling the baseline values), while SG caused a marked decrease. High variability in intestinal ammonium levels has been previously reported in healthy volunteers,^42^ with Martínez-Cuesta et al. demonstrating higher concentrations in severe obesity.^18^ Once again, normal cutoffs need to be established, as well as determining the correlation between serum and feces and their production after food intake. Ammonium is a normal metabolite of amino acid digestion and is one of the most significant toxicants for humans. Hyperammonemia due to defective ammonium detoxification of urea occurs in liver injury, causing severe neurological disorders,^43^ which are successfully treated with antibiotics to reduce the bacterial load in the small intestine. The contribution of gut microbiota to ammonium production is likely underestimated in healthy individuals because it is rapidly converted to urea and excreted. The capacity of intestinal bacteria to overproduce ammonium should, however, not be neglected in various health conditions, as well as in digestive tract surgery. Other metabolites such as cresol, indole, trimethylamine N-oxide and even bile salts can contribute to intestinal and systemic toxicity in patients who undergo bariatric surgery and should be determined in further studies. In fact, the bacterial overgrowth has been previously described after RYGB,^44,45^ being the SG approach recommended for hepatopathy as cirrhosis, but in Nonalcoholic SteatoHepatitis (NASH), RYGB and SG have similar results.^46–48^

We aimed to assess the impact of the preoperative low calorie diet, given that postoperative dietary changes cannot be properly evaluated without the intrinsic effect of the surgery. Bacterial rearrangements were detected in both groups. Al Assal *et al*. reported a significant decrease in *Ruminococcaceae*/*F.prausnitzii* after a low calorie diet.^15^ In our series, however, *F. prausnitzii* decreased only in the SG group, in which, contrary to expected, Bacillota considerably increased, and these variations were not followed by weight loss. Individuals with severe obesity are often undernourished, particularly for essential micronutrients, and recent data have increased the suspicion that the gut microbiota can contribute to this state,^49^ which is a target of interest for future studies to improve postoperative diets.

The DM2 resolved with no drug treatment after the bariatric surgery in 75% of the patients who had this metabolic disorder, which was accompanied by defined changes in intestinal microbial patterns, as previously reported.^7,15,50,51^ The contribution of the intestinal microbiota in resolving DM2 is still controversial, whereas biliary salts appear to play a relevant role.^32^ We were unable to identify a correlation between bacterial genera abundance and DM2 resolution in 75% of our patients (9/12). The baseline status was related to a lower abundance of the Clostridia class, although bariatric surgery promoted a significant decrease in this taxon in all participants.

A robust bioinformatics analysis was incorporated to detect correlations between microbiota composition and functional variables. Despite our efforts, the integrative analysis provided no statistically significant results, and although the Lasso model identified 8 bacterial genera for predicting weight loss, their low representation in the entire ecosystem hinders their use, except for *Bifidobacterium*, which decreased in both surgical groups in direct correlation with weight loss.

At the design stage, all participants were equally considered in their baseline sampling when the surgical technique had not yet been assigned. However, given that each group displayed significant particularities, we decided to analyze the results by considering RYGB and SG separately. Our results strongly suggest different types/degrees of obesity, particularly differentiated by BMI and bacterial metabolism, as has been previously suggested by other authors.^52^ Certainly, a BMI ≥50 implies differential bacterial characteristics compared with lower BMIs, although we cannot ignore the fact that almost all the participants with the highest BMI values underwent RYGB.

Further studies should address the characterization of fecal and serum metabolites from gut microbiota, focusing on their normal ranges to decipher their actual role in obesity and determine the impact of bariatric surgery on the host’s health. Our sampling ended 3 months after surgery, so the long-term effects could not be determined. Although most authors follow patients for a year, the most drastic changes related to gut microbiota appear to occur during the first three months 30].

## Conclussions

Rather than a gut microbiota characteristic of severe obesity, we have detected individual signatures of composition and functionality, with opposing trends for almost all determined variables in the two surgical approaches. RYGB provokes a deeper impact on gut microbiota composition and a more putrefactive metabolism, probably related to biliary acid redistribution. SCFA production is similar in the two groups, with minimal effects from the bariatric process. Lastly, significantly higher ammonium production occurs in RYGB, although its clinical significance is uncertain. Standardized determinations of microbiota metabolites in feces and serum, as well as normality cutoffs, are needed to reach solid conclusions in future studies.

## Supplementary Material

Supplemental Material

## Data Availability

https://www.ncbi.nlm.nih.gov/bioproject/?term=PRJNA639545

## Abbreviations

GABA: gamma-aminobutyric acid; OTU: operating taxonomic unit; SCFAs: short-chain fatty acids; NASH: non-alcoholic steatohepatitis.
