# Supplementary figures and images for: Fecal Metabolome and Bacterial Composition in Severe Obesity: Impact of Diet and Bariatric Surgery
Source: Gut Microbes. 2022 Jul 28;14(1):2106102. doi: 10.1080/19490976.2022.2106102 (PMC9341356; doi:10.1080/19490976.2022.2106102)

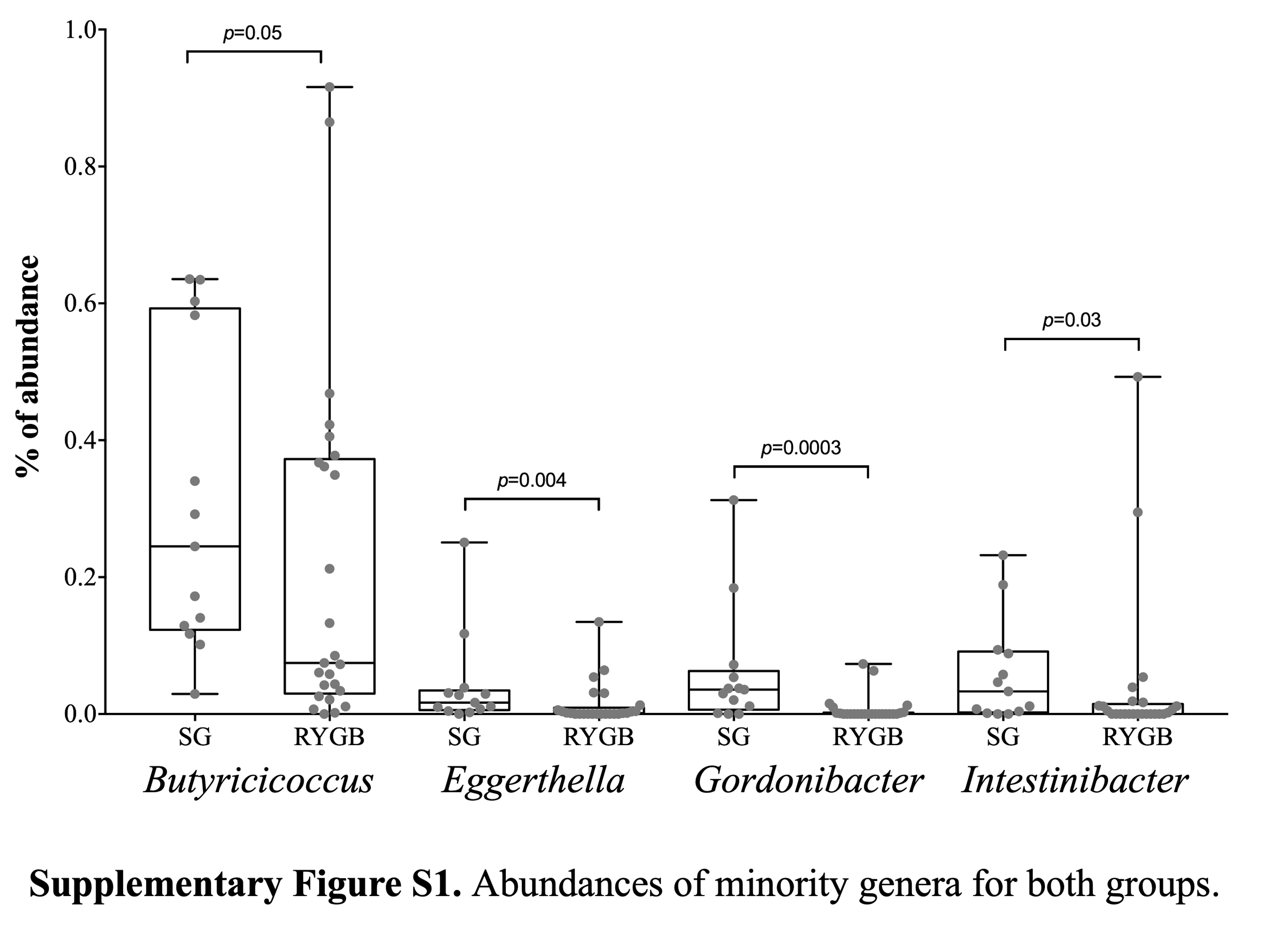

Supplement: Supplemental Material [file KGMI_A_2106102_SM3208.zip › S1.png]

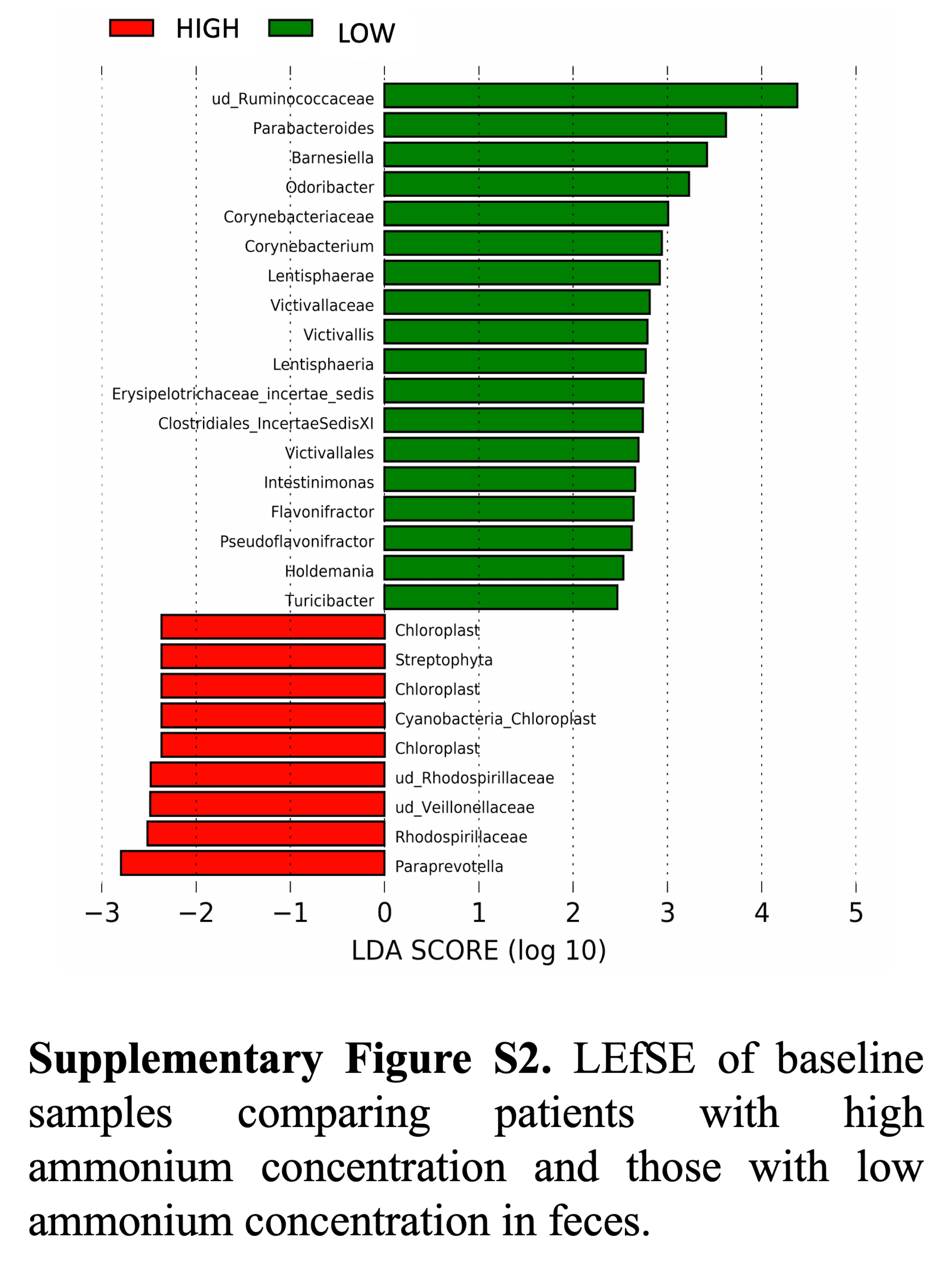

Supplement: Supplemental Material [file KGMI_A_2106102_SM3208.zip › S2.png]

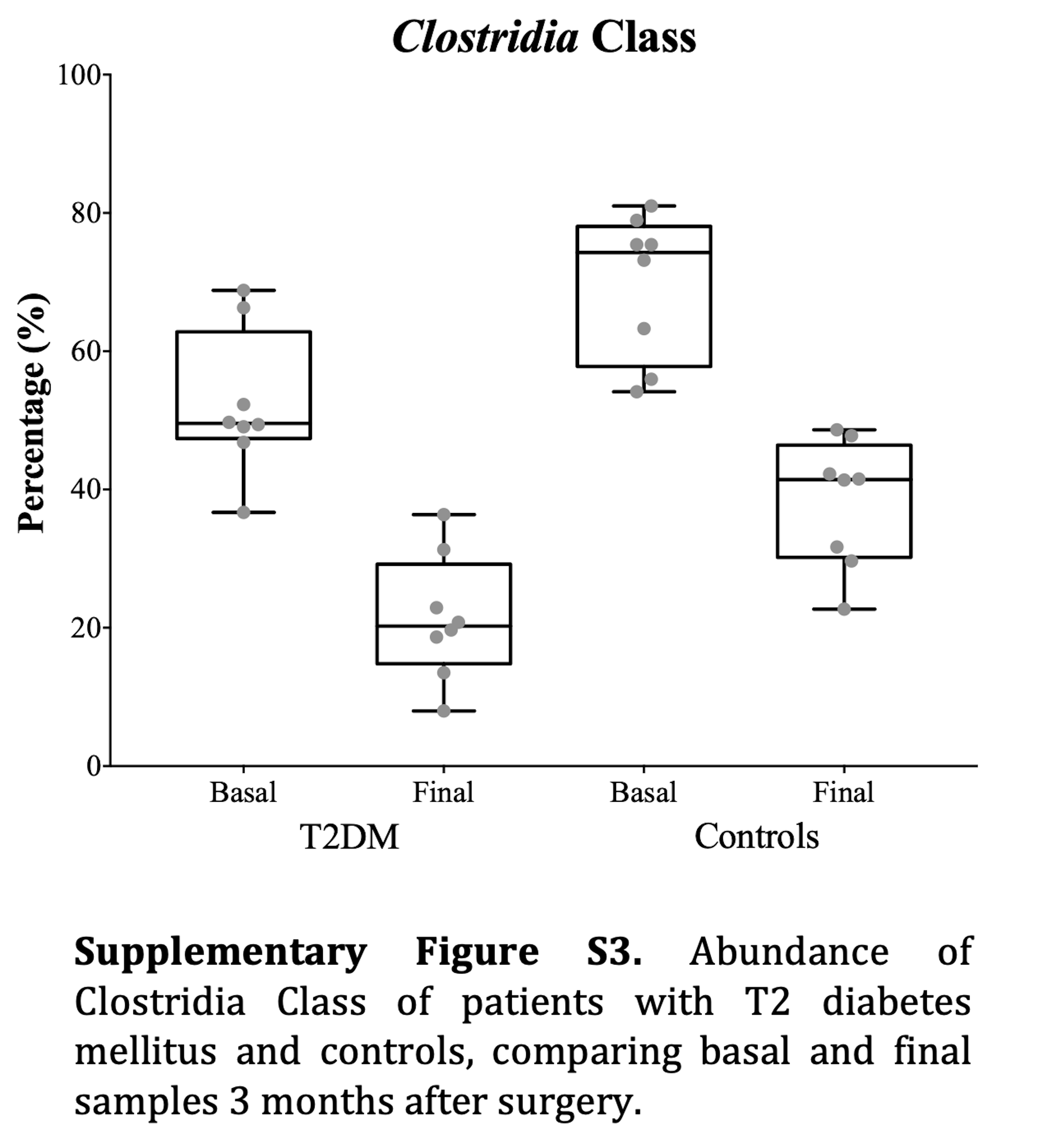

Supplement: Supplemental Material [file KGMI_A_2106102_SM3208.zip › S3.png]
